# Skilled delivery inequality in Ethiopia: to what extent are the poorest and uneducated mothers benefiting?

**DOI:** 10.1186/s12939-017-0579-x

**Published:** 2017-05-16

**Authors:** Brook Tesfaye, Tsedeke Mathewos, Mihiretu Kebede

**Affiliations:** 1 Health Policy and Planning Directorate, Ethiopian Federal Ministry of Health, Sudan Street, Lideta Sub-city, Addis Ababa, Ethiopia; 2 John Snow Incorporated Research and Training Institute, Health Management Information System Scale-up Project, Addis Ababa, Ethiopia; 3 Technical Assistant to Director of Health Policy and Planning Directorate, Ethiopian Federal Ministry of Health, Addis Ababa, Ethiopia; 4 Department of Health Informatics, College of Medicine and Health Sciences, Institute of Public Health, University of Gondar, Gondar, Ethiopia; 5 Leibniz Institute for Prevention Research and Epidemiology – BIPS, Achterstraße 30, Bremen, Germany; 6 University of Bremen, Health Sciences, Bremen, Germany

**Keywords:** Equity, Inequality, Maternal health, Maternal Mortality Ratio (MMR), Universal Health Coverage (UHC), Sustainable Development Goals (SDGs), Developing country, Ethiopia

## Abstract

**Background:**

The fifth Millennium Development Goal (MDG) targeted at improving maternal health. In this regard, Ethiopia has shown substantial progresses in the past two decades. Nonetheless, these impressive gains are unevenly distributed among Ethiopian women with different socio-economic characteristics. This study aimed at investigating levels and trends of skilled delivery service, and wealth and education related inequalities from 2000 to 16.

**Methods:**

Longitudinal data analysis was conducted on Ethiopian Demographic and Health Survey (EDHS) data of 2000, 2005, 2011 and 2016. The outcome variable was skilled delivery, while data on economic status and education level were used as dimensions of inequality. Rate Ratio (RR) and Rate Difference (RD) inequality measures were applied. STATA for windows version 10.1 statistical software was utilized for data analysis and presentation. The strength of association of inequality dimensions with the outcome variable was assessed using a 95% confidence interval.

**Results:**

From total deliveries, 5.62%, 6.3%, 10.8% and 28% of them were attended by skilled birth attendant in 2000, 2005, 2011 and 2016 respectively. In the most recent survey (EDHS 2016), proportion of births attended by skilled birth attendance among women who completed secondary and above education was about 5.42 [95% CI (4.53, 6.09)] times more when compared to women with no formal education. Proportion of births attended by skilled birth attendance among women in the richest quintile was about 5.11 [95% CI (3.98, 6.12)] times higher than that of women in the poorest quintile. Moreover, gap of inequality on receiving skilled delivery service has increased substantially from 24.2 (2000) to 53.8 (2016) percentage points between women in the richest and poorest quintiles; and from 44.9 (2000) to 76.0 (2016) percentage points between women who completed secondary and above education and women with no formal education.

**Conclusions:**

Skilled birth attendance remained low and virtually unchanged during the period 2000–2011, but increased substantially in 2016. Gap on wealth and education related inequalities increased linearly during 2000–16. Most pronounced inequalities were observed in women’s level of education revealing women with no formal education were the most underserved subgroups. Encouraging women in education and economic development programs should be strengthened as part of the effort to attain Universal Health Coverage (UHC) of Sustainable Development Goals (SDGs) in Ethiopia.

## Background

The health status of many populations in developing countries have been substantially improved over the past two decades [1]. Despite the progress achieved so far, Millennium Development Goals (MDGs) particularly the goal of reducing maternal and newborn mortality remains underachieved in many sub-Saharan African countries [2]. Equitable maternal and child health services to improve the health of the women and children across their life course are one of the key priorities of Universal Health Coverage (UHC) [3]. Addressing health service inequalities such as the inequalities between the rich and the poor within one country pose a challenge to policy makers [4].

The MDGs measure health achievements based on aggregated measures of progress. This has masked the inequalities in health outcomes that existed between and within countries and among subgroups in a given population [5]. Learning from the MDGs experience, the 2030 agenda for sustainable development has been firmly anchored in the principle of UHC [6], with a strong commitment to equity [7]. In this context, the implementation of Sustainable Development Goals (SDGs) required metrics to measure inclusion and exclusion of specific population groups [5].

Each year hundreds of thousands of women die due to pregnancy and child birth related causes [8]. Given that the risk of maternal death is highest in 24–48 h of the postpartum period, the presence of skilled birth attendant during childbirth is a key intervention for preventing maternal and newborn deaths. In 2012, about 40 million births in developing countries were not attended by skilled health personnel [9].

Inequalities in maternal health have been widely acknowledged, both across countries [9–11] and within countries [12]. The proportion of births delivered by skilled birth attendant has been identified as the maternal health intervention indicator with the most pronounced economic-related inequality [12].

On this subject, over the past decades, Ethiopia has made great efforts to strengthen its health system and improve the health of women. Ethiopia is one of the few African countries that has reached its target in improving maternal health and reducing child mortality [13]. Despite these encouraging achievments, key child and maternal health services were struck with unfair distribution of maternal health service within and across regions, and across population subgroups based on variety of socio-economic variables [14] such as, between richest versus poorest and most educated versus less educated.

Ethiopia has made substantial progress in improving the health of the population by achieving most of the health-related MDGs [15]. Despite rapid and double-digit economic growth over the past two decades, Ethiopia remains one of the poorest countries in the world [16] with 22% of the people living below the income poverty line [17]. Ethiopia is considered as an example for low-income countries to attain MDGs with limited resource coupled with a sustained political will and commitment to provide innovative policies, strategies and programs [18]. However, analyzing improvements through an equity lens reveals that the rapid economic growth has not been enjoyed fairly across the different segments of the population. In addition, the impressive gains in health sector in recent years are unevenly distributed, and aggregated indicators hide striking inequalities across the population subgroups.

For this reason, the Ethiopian Federal Ministry of Health has designed a new plan, the Health Sector Transformation Plan (HSTP) 2015/16–2019/20, to improve health by addressing inequalities [19]. Inequalities in the main socio-economic stratifiers such as poorest versus richest and less educated versus most educated are major contributors to the overall inequalities in the country. The pervasive inequity among its population, particularly between the poorest and the richest, remains the major health sector challenge.

In the perspective of achieving UHC in a country as diverse as Ethiopia, having large social inequalities combined with fast economic growth, it is clear that inequalities must be measured and trends need to be understood. With policy-makers who are increasingly looking at quantitative evidence to make evidence based decision-making to address health inequalities, monitoring equity and measuring the level of health service inequality is of paramount importance. The purpose of this study is therefore to examine level and trend of skilled delivery service coverage, and wealth and education related inequalities associated with receiving the service in Ethiopia during 2000–16.

## Methods

### Study design and period

Longitudinal data analysis was conducted on Ethiopian Demographic and Health Survey (EDHS) data of 2000, 2005, 2011 and 2016. The study was conducted in Ethiopia from August to December 2016.

### Data sources

The data for this study were retrieved from EDHS data of 200–16. The datasets were main sources to describe key health indicators measuring level of morbidity, mortality and socio-economic progress. Thus, data on skilled delivery with relevant socio-economic characteristics were extracted for this study.

### Sample size and sampling techniques

The Demographic and Health Survey (DHS) program provides household-level data on health, healthcare utilization and ownership of assets for about 60 low and middle income countries in three subsequent periods. The data are based on nationally representative surveys. In most countries, a sample of 5000–10,000 women aged 15–49 years are interviewed to collect data about key health and socioeconomic indicators [20].

The sample was selected using a stratified, two-stage cluster design and Enumeration Areas (EAs) were the sampling units for the first stage. In the most recent survey (EDHS 2016), sample included 624 EAs, 187 in urban areas and 437 in rural areas. A representative sample of 17,067 households were selected for the 2016 EDHS [21].

### Dimensions of inequality

Inequality data and statistics give us an important insight into the state of national economy and the health status of a certain society [22]. To this end, selection of appropriate stratifiers is essential to assess level of inequality from different dimensions [22]. Thus, data on economic status and educational level were used to categorize populations according to dimensions of inequality. These two dimensions of inequality represent common sources of inequality and can be widely applied to populations in low and middle income countries [22]. Economic status is described in terms of a household wealth index which accounts for ownership of certain household items and access to specific services.

On the basis of wealth index, women are categorized into five subgroups; poorest, poorer, medium, richer and richest [21]. Education as a dimension of inequality reflects the highest level of education attained by a mother. Three levels are specified; no education, primary and secondary and above subgroups [22].

### Data processing and analysis

Extracted data were checked for data quality. Ahead of analysis, data disaggregation by wealth and education subgroups was conducted. Disaggregated data shows the level of health inequality in each subgroup of a given dimension of inequality [22].

Simplest inequality measures: absolute inequalities, using Rate Difference (RD) and Relative inequalities, using Rate Ratio (RR) were applied in this study. Relative Concentration Index (RCI) was also utilized to assess level of inequality across subgroups.

STATA for windows version 10.1 statistical software was utilized for data analysis and presentation. The strength of association of inequality dimensions with the outcome variable was assessed using a 95% confidence interval [23]. The results were presented using tables and graphs.

## Results

### Descriptive statistics

In EDHS 2016, a total of 16,515 women in a reproductive age group were included in the study. Out of them, 12, 849 (77.8%) were rural residents. Almost half (47.8%) of the women never attended formal education, while only 2841 (17.2%) completed secondary and above education. The mean age of the women was 27.7 (SD ±9.2), 58.1% of were below the age of 30, reflecting the young age structure of the population. The mean family size of the study population was 5.7 (SD ± 2.68) persons (Table 1).

**Table 1:** Socio-economic characteristics of the mothers in Ethiopia in 2016 (*n* = 16,515)

Characteristics	Frequency	Percentage
Age		
15–29	9656	58.1
30–39	4569	27.3
40–49	2290	13.5
Highest educational level		
No formal education	7894	47.8
Primary	5780	35
Secondary and above	2841	17.2
Wealth status		
Poorest	2842	17.2
Poorer	2956	17.9
Medium	3154	19.1
Richer	3187	19.3
Richest	4376	26.5
Religion		
Orthodox	6986	42.3
Catholic	182	1.1
Protestant	2940	17.8
Muslim	6177	37.4
Others^a^	231	1.4
Place of residence		
Urban	3666	22.2
Rural	12849	77.8
Household size		
Five or less	7944	48.1
Six and above	8571	51.9
Source of water drinking		
Improved^b^	4905	29.7
Unimproved^d^	11610	70.3
Toilet facility		
No facility	5334	32.3
Improved^c^	1040	6.3
Unimproved^e^	8736	52.9

^a^Traditional belief

^b^Piped water into dwelling, Piped water to yard/plot, Public tap or standpipe, Tube-well or borehole, Protected dug well, Protected spring, Rainwater

^c^Flush toilet, Piped sewer system, Septic tank, Flush/pour flush to pit latrine, Ventilated improved pit latrine, Pit latrine with slab, Composting toilet

^d^Water sources not mentioned in improved list

^e^toilet facilities not mentioned in improved list

Only 9.9% of the women gave birth at health facilities for the last 5 years preceding the survey. Six thousand five hundred fifty three percent Six thousand five hundred fifty three percent (39.7%) of the women never gave birth during their life course while 5236 (31.7%) of them had at least one child. Majority of the women (83.7%) never received a family planning service for the last 12 months preceding the survey and only 5789 (35%) visited a health facility for same period. More than half of the women (62.4%) did not have any kind of occupation.

### Level of inequality

**Fig. 1:**
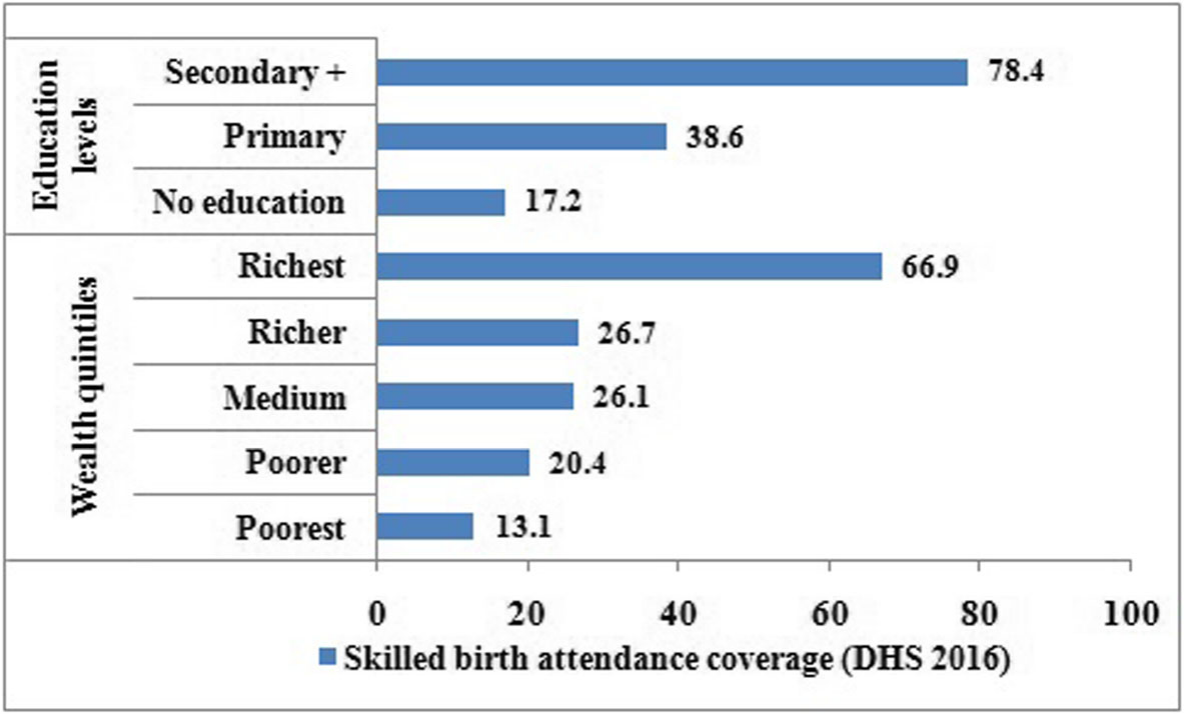
Skilled delivery service disaggregated by economic status and education levels in Ethiopia (EDHS 2016)

**Fig. 2:**
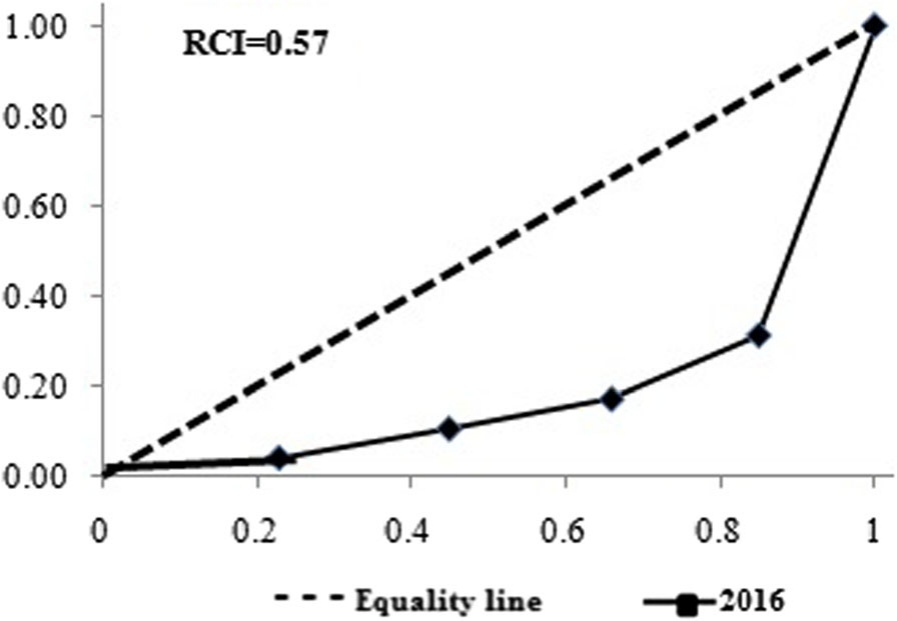
Distribution of the concentration curve disaggregated by economic status in Ethiopia (EDHS 2016)

In the most recent survey (EDHS 2016), skilled delivery service coverage was 28.0%; 13.1% among poorest women subgroup and 66.9% among richest women subgroup (Fig. 1). This implies that proportion of births attended by skilled birth attendant among women in the richest subgroup was about 5.11 [95% CI (4.53, 6.09)] times higher as compared to their poorest counterparts (Table 2). Level of inequality is more pronounced as the concentration curve (Fig. 2) curved downwards with a Relative Concentration Index (RCI) of 0.57 (57%), with women in the richest subgroup accounting for a disproportionately larger fraction of skilled delivery services.

**Table 2:** Skilled delivery inequality by wealth and education characteristics of mothers in Ethiopia during 2000–16 (*n* = 16,515)

EDHS	Inequality dimensions	RD (in percentage points)	SE	95% CI	
2000	Richest vs. poorest	24.23	2.44	19.45	29.01
	Secondary and above vs. no education	44.89	4.94	35.20	54.59
2005	Richest vs. poorest	27.84	2.74	22.47	33.21
	Secondary and above vs. no education	55.37	3.95	47.63	63.11
2011	Richest vs. poorest	47.52	3.45	40.75	54.28
	Secondary and above vs. no education	69.09	4.64	60.00	78.18
2016	Richest vs. poorest	53.81	4.58	47.36	57.19
	Secondary and above vs. no education	51.54	4.76	44.65	55.48

*CI* Confidence Interval, *EDHS* Ethiopian Demographic and Health Survey, *RD* Relative Difference, *SE* Standard Error

**Fig. 3:**
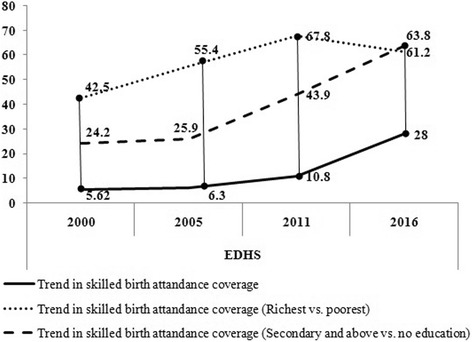
Trends of inequality in skilled delivery service disaggregated by economic status and education in Ethiopia (EDHS 2000–16)

Proportion of births attended by skilled birth personnel also varied significantly across education levels; lowest among women with no formal education (17.2%) and highest among women who completed secondary and above education (78.4%). This implies a 61.2 [95% CI (56.11, 68.08)] percentage point difference (Fig. 3); indicating women who completed secondary and above education received skilled delivery service about 5.42 [95% CI (3.98, 6.12)] times higher as compared to women with no formal education (Table 2).

### Trend of inequality

Proportion of births attended by skilled health personnel remained low and stagnant between 2000 and 11, but showed an exponential increament in 2016 (Fig. 3). Though, the increament was not distributed fairly to women with different socio-economic charachterstics such as between richest versus poorest and most educated versus not educated subgroups.

When disaggregated by economic status, proportion of births attended by skilled health personnel never exceeded five percentage points among women in poorest and poorer subgroups except in 2016. In general, proportion of births attended by skilled health personnel increased by 12.0 percentage points among women in the poorest quintile (from 1.1% in 2000 to 13.1% in 2016) and 18.1% percentage points among women in the poorer quintile (from 1.3% in 2000 to 20.4% in 2016). On the contrary, proportion of births attended by skilled health personnel increased by 23.5 percentage points among women in the richer quintile (from 3.2% in 2000 to 26.7% in 2016) and 41.6% percentage points among women in the richest quintile (from 25.3% in 2000 to 66.9% in 2016).

The data also revealed considerable differences in proportion of births attended by skilled health personnel among women with no formal education (increased from 2.3% in 2000 to 17.2% in 2016) and women who completed secondary and above education (increased from 45.0% in 2000 to 78.4% in 2016) (Fig. 3).

Economic and education related inequalities on receiving skilled delivery service got worsened across the periods. The findings indicate substantially large and consistent inequalities among women in the poorest and richest quintiles (RD: 24.2, 25.9, 43.9 and 63.8 percentage points during 2000, 2005, 2011 and 2016 respectively) (Fig. 3). Similarly, across education subgroups, large and lineraly increasing gaps was observed (Fig. 3) among women with no formal education and women who completed secondary and above education (RD: 42.5, 55.4, 67.8 and 61.2 percentage points during 2000, 2005, 2011 and 2016 resepctively).

## Discussion

The present study aimed to examine levels and trends of proportion of births attended by skilled health personnel, and assess economic and education related inequalities using data from a nationally representative survey. It demonstrated how skilled delivery service and socio-economic related inequalities has arisen between 2000 and 16 and revealed which women subgroups are the most underserved.

Findings of the study revealed that proportion of births attended by skilled health personnel was found to be very low in Ethiopia. Other similar studies from Ethiopia [24, 25] also reported that skilled delivery coverage was low, even as compared to Tanzania and other sub-Saharan African countries [26–28]; 5.62% in 2000, 6.3% in 2005, 10.8% in 2011, and 28% in 2016. The demonstrated increament might be attributed by the Health Extension Program (HEP) [29]. The program delivers healthcare services both at the health post and in the community, with strong fous on sustained preventive health actions and increased health awarness [30]. The health extension workers are expected to provide post-abortion care, family planning, antenatal care (ANC), clean delivery attendance and postnatal care. Furthermore, they are responsible for referring women with obstetric complications to health centers and hospitals where basic and comprehensive emergency obstetric care is available. However, other studies [31–33] have shown the incompetencies of HEWs for managing labour and complications and their incapable role in supporting births [33–35].

The observed findings of the study underscored statistically significant levels of socio-economic inequalities in proportion of births attended by skilled health personnel; most importantly, proportion of births attended by skilled health personnelwas observed to be affected by wealth and education characteristics of women.

The association of education and receipt of skilled delivery was consistent. Women who completed secondary and above education were more likely to deliver with assistance of skilled birth attendant when compared to women with no formal education. This result of the current study is also inline with evidences from Ethiopia [24, 35–41] as well as from other sub-Saharan African countries [24, 28, 41–45]. The effect of education on skilled birth attendance can be explained in a range of ways such as either improving income to spend on healthcare or improving attitude and knowledge towards better healthcare service delivery [46]. Education improves women income and ability to afford the cost of healthcare [47, 48]. Better educated women are also considered to have improved knowledge, attitude and practice of skilled maternity services and benefited in using such services [48–51].

Similarly, our findings also give credence that proportion of births attended by skilled health personnel increased with rising economic status. The findings revealed that women in the poorest quintile typically experienced lower levels of skilled delivery service as compared to their richest counterparts. This is in agreement with findings from previous studies from Ethiopia [52] and other parts of the world [53, 54]. This may be due to the reason that even though skilled delivery services are provided freely in Ethiopia, there may be directly and indirectly associated costs that women in the richest quintile can afford [55]. In broad terms, financial capability of the family and costs of a facility delivery including transportation costs may not be afforded by women among poorest quintile. While directly affecting whether a woman can actually reach a facility for delivery (second delay), the anticipation of high costs will affect whether a decision for a facility delivery is made in the first place (first delay) [44]. Overall, the coverage gap among richest vs. poorest women is the greatest unfairness [56] and, confronts with defined goal of UHC “to ensure that all peoples obtain the health services they need without suffering financial hardship when paying for them” [57, 58].

Results from trend analysis verified that the gap in equity in skilled delivery service has worsened and increased linearly across economic status and education levels. Thus, richest and women who completed secondary and above education are the better off subgroups in receiving skilled delivery service. The observed findings from the inequality analysis are in conformity with what is obtainable in previous studies [59–63].

Overall, the evidence we found suggested that towards universal health coverage for skilled delivery services in Ethiopia is still a long way to go. as majority of poorest and uneducated women do not make use of the available skilled delivery services.

### Limitations of the study

Authors did not apply statistic tests on performing time-trend analysis since number of years considered were few. This study employed secondary data which was primarily collected for DHS objectives. Because of this, we were not able to report on information about other relevant variables such as type of labor (cesarean or natural). Findings of the study did not include variables routinely collected through Health Management Information System (HMIS) reports.

## Conclusions

Skilled delivery service remained low and has shown small increment in Ethiopia during 2000–2011, but showed substantial increment in 2016. Gap on wealth and education related inequalities increased linearly during 2000–16. Most pronounced inequalities were seen by women level of education revealing women with no formal education were the most underserved subgroups. Encouraging mother’s education and economic development programs geared at improving incomes of women should be strengthened. Further studies should be conducted to assess determinants of skilled delivery service and their relative contribution over socio-economic related inequalities as Ethiopia is looking forward in achieving UHC of Sustainable Development Goals (SDGs).

## Abbreviations

EDHS: Ethiopian Demographic and Health Survey
HEP: Health Extension Program
MDGs: Millennium Development Goals
RCI: Relative Concentration Index
RD: Rate Difference
RR: Rate Ratio
SDGs: Sustainable Development Goals
UHC: Universal Health Coverage
